# Copy number variations of circulating, cell-free DNA in urothelial carcinoma of the bladder patients treated with radical cystectomy: a prospective study

**DOI:** 10.18632/oncotarget.17657

**Published:** 2017-05-07

**Authors:** Armin Soave, Felix K.-H. Chun, Timo Hillebrand, Michael Rink, Lars Weisbach, Bettina Steinbach, Margit Fisch, Klaus Pantel, Heidi Schwarzenbach

**Affiliations:** ^1^ Department of Urology, University Medical Center Hamburg-Eppendorf, Hamburg, Germany; ^2^ AJ Innuscreen GmbH, Berlin, Germany; ^3^ Department of Tumor Biology, University Medical Center Hamburg-Eppendorf, Hamburg, Germany

**Keywords:** urothelial carcinoma of the bladder, copy number variation, multiplex ligation-dependent probe amplification (MLPA), cell-free DNA (cfDNA), chromosomal region

## Abstract

The aim of the present study was to establish a rapid profiling method using multiplex ligation-dependent probe amplification (MLPA) and characterize copy number variations (CNV) in circulating, cell-free DNA (cfDNA) in 85 urothelial carcinoma of the bladder (UCB) patients treated with radical cystectomy (RC). MLPA was tested for the use of cfDNA extracted from serum and plasma by various commercial extraction kits. Eighteen probes served as reference to control denaturation, ligation and amplification efficiency. MLPA was exclusively suitable for cfDNA extracted from serum. Serum from 72 patients (84.7%) could be analyzed. Thirty-five patients (48.6%) had presence of CNV in cfDNA. The median CNV count in patients with presence of CNV was 2. Predominantly, CNV were located in the genes CDH1, ZFHX3, RIPK2 and PTEN in 15 patients (20.8%), 12 patients (16.7%), 9 patients (12.5%) and 7 patients (9.7%), respectively. CNV in TSG1, RAD21, KIAA0196, ANXA7 and TMPRSS2 were associated with presence of variant UCB histology (*p* = 0.029, 0.029, 0.029, 0.029, 0.043, respectively). Furthermore, CNV in miR-15a, CDH1 and ZFHX3 were associated with presence of incidental prostate cancer (*p* = 0.023, 0.003, 0.025, respectively). Patients with CNV in KLF5, ZFHX3 and CDH1 had reduced cancer-specific survival, compared to patients without CNV in these genes (pairwise *p* = 0.028, 0.026, 0.044, respectively). MLPA represents an efficient method for the detection of CNV among numerous genes on various chromosomal regions. CNV in specific genes seem to be associated with aggressive UCB biologic features and presence of incidental prostate cancer, and may have a negative impact on cancer-specific survival.

## INTRODUCTION

Urothelial carcinoma of the bladder (UCB) is the second most common genitourinary malignancy and a fatal disease, accounting for 74,000 new cases and 16,000 deaths in the USA in 2015 [1]. For patients with muscle-invasive and recurrent high-risk non-muscle invasive UCB, radical cystectomy (RC) with bilateral pelvic lymphadenectomy represents the golden standard surgical treatment [2]. Despite major improvements in surgical techniques, imaging, perioperative management and systemic chemotherapy, outcomes have remained stable over the past decades [3], and a relevant number of patients will experience disease progression within two years after RC [4]. Various clinico-pathologic UCB features and biomarkers predicting disease recurrence and progression following RC have been introduced [5]; however, none has succeeded in daily clinical practice. Thus, there is still an urgent need for new biomarkers allowing accurate prediction of the true UCB tumor biology, helping to select patients who might best benefit from multimodal treatments and emerging targeted therapy.

Tumor cells and healthy cells release their DNA into the circulatory system. Tumor cell derived circulating cell-free DNA (cfDNA) represents an encouraging blood-based biomarker in various malignancies [6–8] including UCB [9]. UCB is a heterogeneous disease with complex underlying genomic alterations [10], which can be detected in cfDNA during tumor growth and disease progression [11, 12]. Real-time extraction of cfDNA from blood plasma or serum offers the promising opportunity to reveal the molecular UCB biology and course of the disease. In this regard, copy number variations (CNV) comprising DNA amplifications and deletions are a prominent source of genetic variations in cfDNA. Multiplex ligation-dependent probe amplification (MLPA) is a semi-quantitative technique for determining the relative CNV of multiple tumor suppressor genes and oncogenes in a single multiplex PCR-based analysis. To characterize CNV in cfDNA by an easy and rapid method, the aim of the present study was to establish MLPA for the use of cfDNA, together with a data analyses software custom-developed for this assay. In a single reaction, MLPA allows analyzing CNV in 43 chromosomal regions containing 37 genes.

## RESULTS

### Evaluation of the cfDNA extraction from serum and plasma

To test whether MLPA assay is suitable for the analyses of cfDNA in UCB patients, we compared the extractions of cfDNA from plasma and serum using DNA extraction kits from different companies (Qiagen, Macherey Nagel and Analytik Jena). Although cfDNA was additionally precipitated with carrier cfDNA from plasma or serum, we did not get any results on CNV by MLPA using the kits from Qiagen and Macherey Nagel. Exclusively, the PME free-circulating DNA Extraction kit (PME kit, Analytik Jena) was able to provide reliable and solid data on CNV, but only with serum. Adequate amounts of cfDNA could even be precipitated from serum by the PME kit without carrier DNA. With the NanoDrop spectrometer, we measured average concentrations of 700 ng and 100 ng in 1 ml serum and plasma, respectively, indicating that the PME kit is rather suitable for serum than plasma and the failure of the performance of MLPA using cfDNA extracted from plasma with the PME kit. Due to the supplementation of carrier DNA, the amounts of cfDNA extracted from serum or plasma with the kits from Qiagen and Macherey Nagel could not unambiguously determined. For all further experiments with the MLPA assay, we used the PME kit and at least 50 ng cfDNA (in 5 μl solution buffer) extracted from serum. Supplementary Figure 1 shows exemplary analyzable and unanalyzable electropherograms derived from capillary electrophoresis using the PME kit and the QiAmp DNA Blood Mini kit, respectively.

### Clinico-pathologic UCB characteristics according to the CNV status

**Figure 1:**
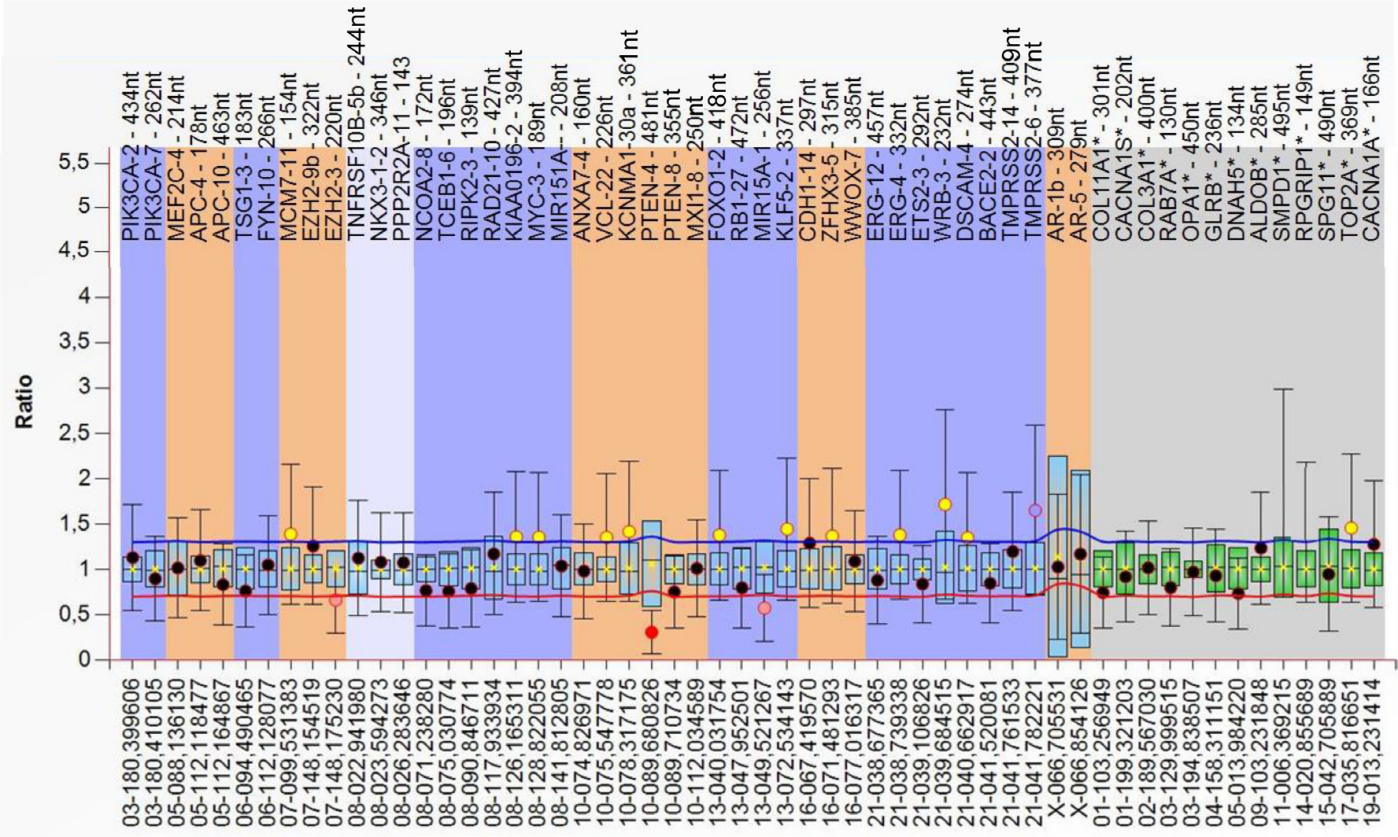
The box plot shows data of an exemplary serum sample (as calculated by Coffalyser.Net software) The DNA probes are arranged by chromosomal locations. The target-specific probes have a blue, orange and white background (left), whereas the reference probes have a grey background (right). Red point indicates a significant decrease in CNV, whereas yellow/orange points are ambiguous. A reduced copy number is clearly detected in PTEN. The data were calculated by intra- and inter-sample comparisons. Intra-sample normalization was performed by dividing the fluorescence signal of each target-specific probe by the signal of every single reference probe in this probe. The median of all these ratios of this probe is the normalization constant. Subsequently, inter-sample comparison was performed by dividing the normalization constant of each probe of this sample by the average normalization constant of all 72 reference (leukocyte) samples.

Using the cfDNA and genomic DNA (negative control) extracted from serum and the corresponding leukocytes of each patient, respectively, by the PME kit, we analyzed 43 chromosomal regions containing 37 genes for CNV by the MLPA assay. The CNV status was defined as the presence of DNA amplifications (DNA gains) or DNA deletions (DNA losses). As expected from normal, wild type (wt) DNA, genomic leukocyte DNA was analyzable in all 85 UCB patients and did not show any CNV, thus no genomic variations. Serum cfDNA samples were analyzable in 72 patients (85.0%) out of the 85 UCB patients. Due to the low serum DNA amounts, 13 serum samples could not be analyzed. In total, 35 out of the 72 analyzable patients (48.6%) had presence of CNV in cfDNA. The median CNV count in patients with presence of CNV was 2 (mean: 2.4; standard deviation: 1.4; range: 1–6). Most CNV were located in the genes CDH1, ZFHX3 (both copy number gains in chromosome 16), RIPK2 (copy number losses in chromosome 8) and PTEN (copy number losses in chromosome 10) in 15 patients (20.8%), 12 patients (16.7%), 9 patients (12.5%) and 7 patients (9.7%), respectively (Supplementary Table 2). Figure 1 shows a box plot of an exemplary CNV analysis in a serum sample. The CNV status was not associated with any clinico-pathologic UCB feature (Table 1). However, CNV in TSG1, RAD21, KIAA0196, ANXA7 and TMPRSS2 were associated with presence of squamous and non-squamous cell differentiation variant UCB histology (all *p ≤* 0.043; Table 2). In addition, CNV in miR-15a, CDH1 and ZFHX3 were associated with presence of incidental prostate cancer in the RC specimen (all *p*-values ≤ 0.025; Table 2).

**Table 1:** Descriptive characteristics of 72 urothelial carcinoma of the bladder patients treated with radical cystectomy and bilateral lymphadenectomy stratified by the copy number variations status

	All (*n* =72)	CNV status negative (*n* = 37)	CNV status positive (*n* = 35)	*p*-value
Age [year; median (IQR)]	70.4 (60.5; 74.8)	70.2 (64.9; 75.9)	70.1 (56.5; 73.4)	0.123***
Gender (*n*; %)				
Male	57 (79.2)	27 (73.0)	30 (85.7)	0.249*
Female	15 (20.8)	10 (27.0)	5 (14.3)	
Clinical tumor stage (*n*; %)				
cTa, cTis	4 (5.6)	3 (8.1)	1 (2.9)	
cT1	20 (27.8)	11 (29.7)	9 (25.7)	0.642**
cT2	45 (62.5)	21 (56.8)	24 (68.6)	
cT3	3 (4.2)	2 (5.4)	1 (2.9)	
Clinical tumor grade (*n*; %)				
cG1	1 (1.4)	0 (0)	1 (2.9)	0.582**
cG2	6 (8.3)	3 (8.1)	3 (8.6)	
cG3	65 (90.3)	34 (91.9)	31 (88.6)	
Intravesical chemo- and/or immunotherapy prior to RC (*n*; %)				
No	56 (77.8)	28 (75.7)	28 (80.0)	0.779*
Yes	16 (22.2)	9 (24.3)	7 (20.0)	
Number of TURB prior to RC [median (IQR)]	1 (1; 2)	1 (1; 3)	1 (1; 2)	0.355***
Days between last TURB and RC [median (IQR)]	39 (27; 61)	49 (28; 79)	36 (25; 50)	0.050***
Pathologic tumor stage (n; %)				
pT0, pTa, pTis	21 (29.2)	8 (21.6)	13 (37.1)	0.260**
pT1	5 (6.9)	4 (10.8)	1 (2.9)	
pT2	20 (27.8)	10 (27.0)	10 (28.6)	
pT3	15 (20.8)	7 (18.9)	8 (22.9)	
pT4	11 (15.3)	8 (21.6)	3 (8.6)	
Combined tumor stage (n; %)				
Localized (pT≤2)	46 (63.9)	22 (59.5)	24 (68.6)	0.469*
Advanced (pT3-4)	26 (36.1)	15 (40.9)	11 (31.4)	
Combined disease stage (n; %)				
≤ pT2 and pN0	43 (59.7)	21 (56.8)	22 (62.9)	0.637*
≥ pT3 or pN1-3	29 (40.3)	16 (43.2)	13 (37.1)	
Pathologic tumor grade (n; %)				
G2	2 (2.8)	6 (16.2)	7 (20.0)	0.764*
G3	59 (81.9)	31 (83.8)	28 (80.0)	
Concomitant carcinoma *in situ* (n; %)				
Absent	50 (69.4)	26 (70.3)	24 (68.6)	0.999*
Present	22 (30.6)	11 (29.7)	11 (31.4)	
Lymphovascular invasion (n; %)				
Absent	53 (73.6)	26 (70.3)	27 (77.1)	0.597*
Present	19 (26.4)	11 (29.7)	8 (22.9)	
Micro-vessel invasion (n; %)				
Absent	65 (90.3)	33 (89.2)	32 (91.4)	0.999*
Present	7 (9.7)	4 (10.8)	3 (8.6)	
Lymph node status (n; %)				
pN0	54 (75.0)	25 (67.6)	29 (82.9)	0.177*
pN1-3	18 (25.0)	12 (32.4)	6 (17.1)	
Number of lymph nodes removed [median, (IQR)]	13 (9; 19)	13 (7; 20)	15 (9; 19)	0.536***
Soft tissue surgical margin status (n; %)				
Negative	61 (84.7)	29 (78.4)	32 (91.4)	0.191*
Positive	11 (15.3)	8 (21.6)	3 (8.6)	
Urothelial carcinoma histology (n; %)				
Pure UCB	53 (73.6)	29 (78.4)	24 (68.6)	0.629**
Presence of squamous cell differentiation	9 (12.5)	4 (10.8)	5 (14.3)	
Presence of non-squamous cell differentiation	10 (13.9)	4 (10.8)	6 (17.1)	
Presence of incidental prostate cancer in the RC specimen (n; %)				
No	35 (48.6)	22 (59.5)	13 (37.1)	0.065*
Yes	37 (51.4)	15 (40.5)	22 (62.9)	
Adjuvant chemotherapy (n; %)				
Not administered	53 (73.6)	26 (70.3)	27 (77.1)	0.597*
Administered	19 (26.4)	11 (29.7)	8 (22.9)	
Adjuvant chemotherapy regimen (n; %)				
Cisplatin-based	11 (57.9)	7 (63.6)	4 (50.0)	0.676**
Carboplatin-based	8 (42.1)	4 (36.4)	4 (50.0)	

CNV status negative: no DNA amplifications or deletions.

CNV status positive: presence of DNA amplifications or deletions.

* = Fisher's Exact test.

** = Pearson χ^2^ test.

*** = Mann-Whitney-*U* test.

Abbreviations: CNV = copy number variations; IQR = interquartile range; RC = radical cystectomy; TURB = transurethral resection of the bladder.

**Table 2:** Urothelial carcinoma histology and presence of incidental prostate cancer in the radical cystectomy specimen of 72 urothelial carcinoma of the bladder patients treated with radical cystectomy and bilateral lymphadenectomy stratified by copy number variations of selected genes

	**TSG1**		***p*-value**	**KIAA0196**		***p*-value**	**RAD21**		***p*-value**	**ANXA7**		***p*-value**	**TMPRSS2**		***p*-value**
	**No CNV**	**Dec**		**No CNV**	**Inc**		**No CNV**	**Inc**		**No CNV**	**Inc**		**No CNV**	**Inc**	
Urothelial carcinoma histology (*n*; %)			0.029			0.029			0.029			0.029			0.043
Pure UCB	53(74.6)	0(0)		53(74.6)	0(0)		53(74.6)	0(0)		53(74.6)	0(0)		53(74.6)	0(0)	
Presence of squamous cell differentiation	8(11.3)	1(100.0)		8(11.3)	1(100.0)		8(11.3)	1(100.0)		8(11.3)	1(100.0)		9(12.7)	0(0.0)	
Presence of non-squamous cell differentiation	10(14.1)	0(0.0)		10(14.1)	0(0.0)		10(14.1)	0(0.0)		10(14.1)	0(0.0)		9(12.7)	1(100.0)	
	**miR-15a**		***p*-value**	**CDH1**		***p*-value**	**ZFHX3**		***p*-value**						
	**No CNV**	**Dec**		**No CNV**	**Inc**		**No CNV**	**Inc**							
Presence of incidental prostate cancer in the RC			0.023			0.003			0.025						
specimen (*n*; %)	30(44.8)	5(100.0)		33(57.9)	2(13.3)		33(55.0)	2(16.7)							
No	37(55.2)	0(0.0)		24(42.1)	13(86.7)		27(45.0)	10(83.3)							
Yes															

Abbrviations: ANXA7 = annexin A7; CDH10 = E-cadherin; CNV = copy number variations; Dec = decreased (copy number loss); Inc = increased (copy number gain); KIAA0196 = alias spastic paraplegia 8 (SPG8); miR-15a = microRNA 15a; RAD21 = radiation-sensitive 21; RC = radical cystectomy; TMPRSS2 = transmembrane protein serine kinase; TSG1 = tumor suppressor gene 1; ZFHX3 = zinc finger homebox 3.

Dec, decreased (DNA deletion, copy number loss).

Inc, increased (DNA amplification, copy number gain).

The *p*-valuss are derived fron the Fisher's Exact test.

### Outcomes according to the CNV status

The median follow-up of cancer sur*vivo*rs was 16 months (IQR: 4; 28). Actuarial two-year recurrence-free, cancer-specific and overall survival estimates were 67% ± 7% (standard error), 92% ± 4% and 88% ± 5% respectively.

**Figure 2:**
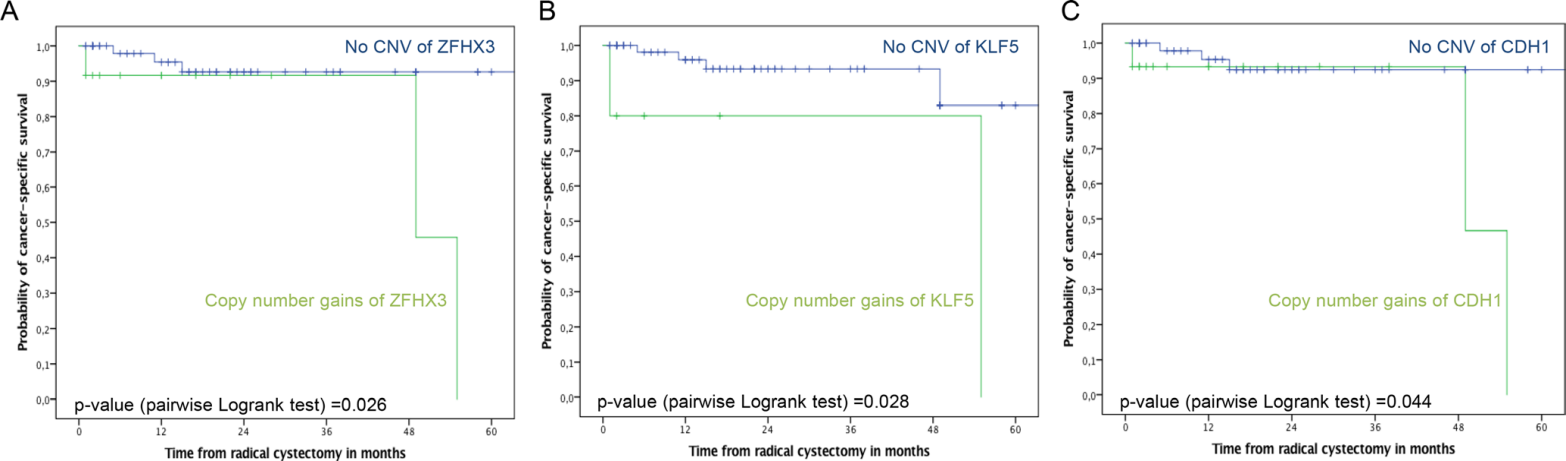
Kaplan-Meier plots of cancer-specific survival stratified by CNV in ZFHX3 (**A**), KLF5 (**B**) and CDH1 (**C**) in 72 UCB patients treated with RC and bilateral pelvic lymphadenectomy. Top curves show UCB patients with no CNV (no genomic aberrations), and bottom curves show patients with CNV comprising DNA amplifications.

In Kaplan-Meier analyses there was no difference in recurrence-free, cancer-specific and overall survival according to the CNV status in cfDNA (pairwise *p* = 0.409, 0.419 and 0.477, respectively; Figure not shown). However, patients with copy number gains in KLF5, ZFHX3 and CDH1 had reduced cancer-specific survival, compared to patients without CNV in these genes ZFHX3 and CDH1, respectively (pairwise *p* ≤ 0.044; Figure 2). In univariable logistic regression analysis, copy number gains in KLF5 (hazard ratio (HR): 3.2; 95% confidence interval (CI): 1.2–9.1; *p* = 0.025) and ZFHX3 (HR: 2.5; 95%CI: 1.1–6.0; *p* = 0.038) were associated with cancer-specific mortality.

## DISCUSSION

In the current study, we tested whether the MLPA assay is suitable for the analyses of cfDNA in blood of UCB patients. In contrast to the high amounts of intact genomic DNA derived from cells, plasma/serum frequently delivers insufficient amounts of cfDNA that is additionally fragmented. These limitations of getting adequate amounts of qualitatively good cfDNA and the additional prevalence of wild-type cfDNA over tumor cfDNA in blood indicates the difficulties to establish reliable multiplex genetic analyses with tumor derived cfDNA. With a specific extraction method, i.e., the PME kit and provided that at least 50 ng of cfDNA can be recovered from this kit, we found CNV in serum cfDNA from nearly each second UCB patient. The reliability of the CNV detection was examined by a repetitive analysis of some serum cfDNA samples and by the fact that genomic leukocyte DNA did not show any CNV in all 85 UCB patients.

Currently, it still remains a matter of debates, whether serum or plasma is the optimal source for cfDNA analyses [13]. However, within the framework of our study, our findings indicate that serum is only suitable for the MLPA assay. The lack of getting data with plasma is not based on the cfDNA amounts and may rather be due to anticoagulants required for the preparation of plasma, such as heparin, acid citrate dextrose (ACD) or EDTA. These agents may affect the MLPA [14]. In addition, preanalytical variables [13] may also affect this assay. However, their description is beyond the scope of the present study. For the extraction of cfDNA from plasma and serum, we used various established commercial extraction kits, which are commonly applied by numerous research groups. Notably, only the PME cfDNA Extraction kit was suitable for CNV analyses with the MLPA assay. Possibly, the components of the other extraction kits may have an influence on the performance of MLPA. In this context, it has also been demonstrated that the NucleoSpin Plasma XS Kit from Macherey-Nagel [15] is superior to the QIAamp DNA blood mini kit [16–18] in terms of yield, purity and efficiency of small DNA fragment retrieval. Likewise, the NucleoSpin Plasma XS Kit did also not provide data with the MLPA assay. Therefore, further studies are needed to shed more light on the important issue of differences among various cfDNA extraction methods from serum.

To carry out the MLPA assay using serum cfDNA, it should also be considered that tumor-derived cfDNA is diluted by wild type cfDNA in the blood of cancer patients that may camouflage the detection of genetic alterations in tumor-derived cfDNA. Therefore, the establishment of a reliable detection method of CNV in cfDNA is still challenging. Although the low prevalence of tumor-derived cfDNA is a drawback of carrying out such analyses [6, 19] we could efficiently detect CNV in cfDNA. MLPA overcomes even the limitations of *in situ* hybridization with its limited resolution of longer than 20 kb DNA molecules which is not suitable for detection of exon-length CNV. Furthermore, the analysis of *in situ* hybridization assays is labor-extensive, and cannot be scaled to high-throughput and -multiplex testing [20].

In respect to our data, the majority of CNV were located in oncogenes and tumor suppressor genes, including CDH1, ZFHX3, RIPK2 and PTEN. These genes play a major role in cancer progression in various malignancies, including bladder cancer [21–24]. For the first time, our findings suggest that CNV in these genes may be of particular relevance for the understanding of the UCB biology. We found that almost half of patients had presence of incidental prostate cancer in the RC specimen, which is in line to findings of previous studies [25]. The presence of incidental prostate cancer was associated with CNV in miR-15a, CDH1 and ZFHX3. We detected copy number gains in CDH1 and ZFHX3 on chromosome 16, and copy number losses in miR-15a on chromosome 13. Similarly, using comparative genomic hybridization, other authors have suggested that gains on chromosome 16 and losses on chromosome 13 may be common findings in non-metastatic and metastatic prostate cancer [26]. CDH1 is a cadherin cell adhesion molecule that is involved in epithelial-mesenchymal transition [21]. Using RNA-Seq and real-time qPCR, CDH1 was shown to be up-regulated in recurrent muscle-invasive cisplatin-resistant UCB tissue compared with adjacent non-tumor tissue [27]. Interestingly, genomic gains in the transcription factor ZFHX3 could also be found in circulating tumor cells from prostate cancer patients [28]. Moreover, the putative tumor suppressor miR-15a was described to be homozygously deleted in prostate cancer cell lines and xenografts [29]. Expression of this microRNA inhibited cell proliferation, promoted apoptosis of cancer cells, and suppressed tumorigenicity of diverse cancer types, both *in vitro* and *in vivo* [30].

Transformation from pure UCB to variant UCB histology has been suggested being a loss of differentiation [31]. Still, genetic and epigenetic modifications during the transformation from pure UCB to variant UCB histology remain mainly unresolved [31, 32]. In this regard, we found CNV in specific genes, such as TSG1, RAD21, KIAA0196, ANXA7 and TMPRSS2, to be associated with the presence of variant UCB histology at RC. RAD21 encodes a key component of the cohesin complex, which is essential for chromosome segregation, and RAD21 deregulation may impact survival in breast cancer [33]. TSG1 and KIAA0196 are poorly characterized genes. In prostate cancer, KIAA0196 is amplified and associated with poor prognosis [34]. The cancer-specific expression of ANXA7, a GTPase, has been described as a diagnostic marker of cancer and a potential target for cancer treatment. Cross talk of ANXA7 with PTEN and EGF receptor led to constitutive activation of PI3K-AKT signaling, a central pathway of tumor cell survival and proliferation [35]. To sum up, our findings suggest that genetic instability in variant histology may result in CNV in specific genes. However, further studies with larger patient cohorts with variant UCB histology are needed to verify this hypothesis.

We found that the CNV status was not associated with outcomes. However, patients with CNV in specific genes, such as ZFHX3, KLF5 and CDH1, had reduced cancer-specific survival compared to patients without CNV in these genes. Patients with copy number gains in KLF5 and ZFHX3 were at a 3.2-fold and 2.5-fold increased risk of cancer-specific mortality, respectively. Previously, it was reported that the transcription factor KLF5 is involved in tumorigenesis of UCB, by promoting cell proliferation, migration and angiogenesis [36–38]. Thus, KLF5 could become a promising therapeutic target molecule for UCB. Recently, in an established xenograft mouse model of colon cancer, the drug ML264 efficiently inhibited growth of the tumor within 5 days of treatment by inhibiting the expression of KLF5 and EGR1, a transcriptional activator of KLF5 [39]. Moreover, in non-muscle invasive UCB treated with TURBT, the tumor suppressive transcription factor ZFHX3 seems to be an independent predictor for disease recurrence [22].

To our knowledge this is the first study investigating CNV in cfDNA using MLPA in UCB patients treated with RC, however, it is not devoid of some limitations. First and foremost, overall sample size and follow-up data are limited. Therefore, we cannot exclude that findings may be different in larger patient cohorts with extended follow-up. In addition, the limited number of events, that could also be caused by the prevalence of normal wild type cfDNA in blood of UCB patients, impeded multivariable analysis of risk factors for disease recurrence and survival. Nevertheless, our study remains the first and currently largest using MLPA in UCB patients treated with RC. Approximately every second patient had an incidental prostate cancer in the RC specimens. Thus, CNV in cfDNA detected in our study may be derived from prostate or bladder cancer cells, as well as from both. Further investigations on CNV in the primary tumor together with CNV in cfDNA are necessary to shed light on the source of cfDNA, whether it stems from the primary tumor, circulating tumor cells or micrometastases. A further limitation of our study is the fact that no single analyses on each chromosomal region by real-time Taqman PCR have been performed due to the insufficient serum cfDNA amounts extracted from our patient cohort. However, these analyses concerns future studies, since the main focus of the present study was to establish a quick technique without excessive statistical efforts to reliably analyze multiply CNV in cfDNA.

MLPA represents a simple and efficient method for the detection of CNV among numerous genes on various chromosomal regions. Prior to RC, approximately half of UCB patients harbor CNV in different tumor suppressor genes and oncogenes. CNV in specific genes are associated with aggressive UCB biologic features and presence of incidental prostate cancer in the RC specimen. In addition, CNV in specific genes may have a negative impact on cancer-specific survival. The inclusion of MLPA in future studies is recommended to validate our findings in larger patient cohorts.

## MATERIALS AND METHODS

### Patient cohort

After written informed consent, we prospectively enrolled 85 UCB patients treated with RC and bilateral pelvic lymphadenectomy without neoadjuvant chemotherapy at the University Medical Center Hamburg-Eppendorf between 2011 and 2014. Recurrent Ta, T1, or carcinoma *in situ* (CIS), refractory to transurethral resection of the bladder (TURBT) with or without intravesical immunotherapy or chemotherapy, or muscle invasive UCB were indications for RC. Preoperative staging consisted of computed tomography (CT) of the thorax and abdomen/pelvis, and bone scan and cranium imaging when clinically indicated. Exclusion criteria included metastatic disease at preoperative staging, a history of any other malignancy, previous systemic chemotherapy or radiation, as well as incomplete clinico-pathologic or follow-up data. In total, 19 patients (26.4%) received adjuvant chemotherapy based on tumor stage, overall health status, renal function and patients’ desire. Adjuvant chemotherapy was consistently platinum-based and generally started within 90 days after RC. The study was approved by the local ethics committee (No. PV3962).

### Pathological evaluation

The complete surgical RC specimen was inked, and multiple sections were obtained from the bladder and the tumor in addition to the regional lymph nodes and ureters. Tumor stage and nodal status were assessed according to the tumor, lymph node and metastasis (TNM) system. Tumor grade was assessed according to the 1998 World Health Organization (WHO) grading system [40]. Concomitant CIS was defined as the presence of CIS in conjunction with another tumor other than CIS alone. Lymphovascular invasion (LVI) was defined as the unequivocal presence of tumor cells within an endothelium-lined space without underlying muscular walls [41]. Micro-vascular invasion (MVI) was defined as the presence of tumor cells within a vessel with a vascular wall and red blood cells in the lumen [42]. A positive soft tissue surgical margin (STSM) was defined as the presence of tumor at inked areas of soft tissue on the RC specimen [43]. Presence of variant UCB histology was defined as the presence of UCB combined with any variant histology. Variant UCB histologies were classified corresponding to the WHO Classification of Tumors [44]. Incidental prostate cancer was defined as presence of prostate cancer in the RC specimens [25].

### cfDNA extraction

Preoperative blood samples were usually collected on the day prior to RC at a median of 39 days [interquartile range (IQR): 27; 61] after the preceding TURB. Serum and plasma were prepared from 6 ml whole blood. cfDNA was extracted from serum and plasma using diverse DNA extraction kits (i.e., QiAmp DNA Blood Mini kit, Qiagen, Hilden, Germany; QiAmp Circulating Nucleic Acid kit, Qiagen; NucleoSpin Plasma XS kit, Macherey Nagel, Düren, Germany; PME free-circulating DNA Extraction kit, Analytik Jena, Germany). cfDNA was extracted from 2 ml serum or plasma as well as leukocytes (reference) from 6 ml EDTA blood, and performed according to the manufacturer´s instructions. Quantification and quality of the extracted cfDNA were determined spectrophotometrically using the NanoDrop Spectrometer ND-1000 (Thermo Fisher Scientific, Wilmington, DE, USA).

### MLPA assay

CNV were determined using 5 μl (50 ng) cfDNA, 5 μL (100 ng) leukocyte (reference) DNA from 85 BCa patients and the SALSA MLPA probemix X049-A1 kit (MRC Holland, Amsterdam, The Netherlands). This kit contains a probe mix of 43 sequences of 37 genes to be analyzed and 22 reference genes (Supplementary Table 1). According to the manufacturer's instructions, the MLPA probes were hybridized and ligated to denaturated serum, plasma and leukocyte DNA. During the subsequent PCR, all ligated probes were amplified simultaneously using the same PCR primer pair, of which one PCR primer was fluorescently labelled. Fragment separation was done by capillary electrophoresis on an automated ABI 3130 DNA analyzer (Applied Biosystems, Freiburg, Germany), yielding a specific electropherogram.

### Data normalization

Data normalization was carried out by Coffalyser.Net analysis software (wwww.mlpa.com). It consists of 2 steps: intra- and intersample normalization. For intrasample normalization, within each sample, each probe peak was compared with the peaks of the reference probes. Reference probes located on various chromosomes detect sequences that are expected to have a normal copy number (CN) in all samples. The determined relative probe signals were then used for intersample normalization (Supplementary Table 1). Final probe ratios were determined by comparing the relative probe peak in the cfDNA sample of interest with those of all leukocyte DNA samples. Leukocyte DNA samples are expected to have a normal CN for both the reference and target probe. To avoid false positive data due to the quality and quantity of the serum cfDNA, only unambiguous values were used (Figure 1), and PCR was repeated.

### Follow-up regimen

For the first year following RC, patients were generally seen every three months, from the second to fifth years every six months, and annually thereafter. Follow-up comprised a history, serum chemistry evaluation and physical examination. Diagnostic imaging of the abdomen including the urinary tract (e.g. ultrasonography and/or intravenous urography, CT of the abdomen/pelvis with intravenous contrast) and chest radiography were performed at least annually or when clinically indicated. Further radiographic evaluations (i.e., bone or brain scans, magnetic resonance imaging) were conducted at the discretion of the treating physician when clinically indicated.

Disease recurrence was defined as local failure in the operative site, regional lymph nodes, or distant metastasis. Upper tract urothelial carcinoma was not considered as disease recurrence but metachronous tumor. Cancer-specific mortality was defined as death from UCB. Overall mortality was defined as death from any cause. The cause of death was determined by the treating physician, by chart review corroborated by death certificates, or by death certificates alone [45]. Perioperative mortality (i.e., death within 30 days of surgery) was censored at time of death for bladder cancer-specific survival analyses.

### Statistical analyses

The co-primary endpoints of the present study were disease recurrence, cancer-specific and overall mortality according to CNV in multiple tumor suppressor genes and oncogenes. The indicator variables (i.e., CNV) were analyzed as categorical variables. Associations between categorical variables were assessed using the Fisher exact and χ^2^-test. Differences in continuous variables were analyzed using the Mann-Whitney-*U* test (two categories) and the Kruskal-Wallis test (three or more categories). Recurrence-free, cancer-specific and overall survival probabilities were estimated using the Kaplan-Meier method and differences between groups were assessed using the Log rank statistic. Univariable Cox regression models assessed time to disease recurrence, cancer-specific and overall mortality. All tests are two-sided and a *p-value* of < 0.05 was set to be statistically significant. All analyses were performed with SPSS 20 (SPSS Inc., IBM Corp., Armonk, NY).

## SUPPLEMENTARY MATERIALS FIGURES AND TABLES






