# Supplementary material for: Copy number variations of circulating, cell-free DNA in urothelial carcinoma of the bladder patients treated with radical cystectomy: a prospective study
Source: Oncotarget. 2017 May 7;8(34):56398–407. doi: 10.18632/oncotarget.17657 (PMC5593570; doi:10.18632/oncotarget.17657)
Supplement: Supplementary file 1 [file oncotarget-08-56398-s001.pdf]

Copy number variations of circulating, cell-free DNA in urothelial carcinoma of the bladder patients treated with radical cystectomy: a prospective study

Supplementary Materials

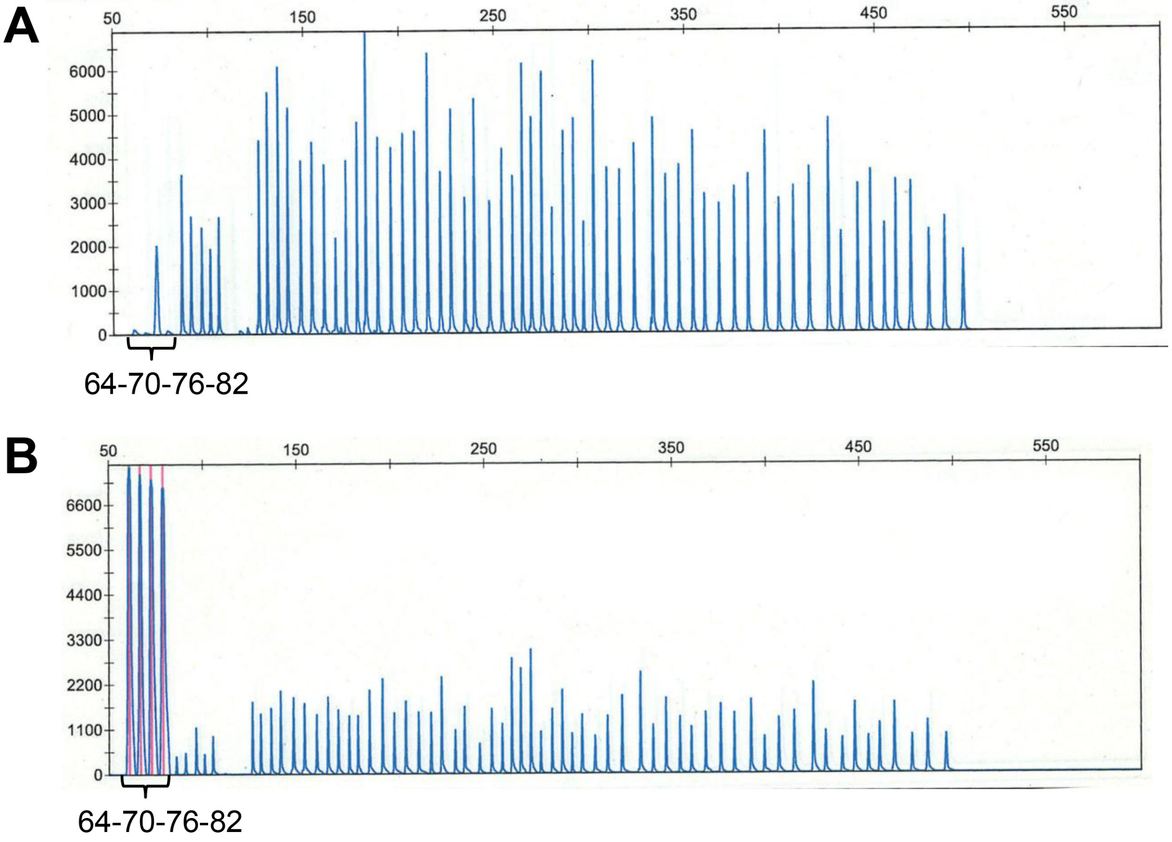

Supplementary Figure 1: Electropherograms derived from capillary electrophoreses. Each probe in an MLPA probemix has a unique amplicon length, typically ranging between 130–500 nt. The first four peaks (quantity fragments, at 64-70-76-82 nt) are complete fragments that do not need to hybridize to DNA or be ligated to be amplified during PCR. The more sample DNA is added, the lower they get. If these peaks are higher than the subsequent probe peaks, then the diagram is not evaluable. (**A**) analyzable and (**B**) unanalyzable experiment.

Supplementary Table 1: SALSA MLPA X049-A1 probe mix, listed by lengths. See Supplementary_Table_1

Supplementary Table 2: Summary of urothelial carcinoma of the bladder patients harboring copy number variations in serum cfDNA. See Supplementary_Table_2
