# Supplementary material for: Copy number variations of circulating, cell-free DNA in urothelial carcinoma of the bladder patients treated with radical cystectomy: a prospective study
Source: Oncotarget. 2017 May 7;8(34):56398–407. doi: 10.18632/oncotarget.17657 (PMC5593570; doi:10.18632/oncotarget.17657)
Supplement: Supplementary file 2 [file oncotarget-08-56398-s002.docx]

**Table S1:** SALSA MLPA X049-A1 probe mix, listed by lengths

| **Functions** | **Genes** | **Chromosomal band** | **Exon** | **Length** |
| --- | --- | --- | --- | --- |
| quantity control | Q-64 |  |  | 64 |
| quantity control | Q-70 |  |  | 70 |
| quantity control | Q-76 |  |  | 76 |
| quantity control | Q-82 |  |  | 82 |
| denaturation control 1 | CARM1 | 19p13.2 | 1 | 88 |
| ligation control | IL1B | 02q13 | 4 | 92 |
| denaturation control 2 | JPH3 | 16q24.2 | up | 96 |
| X presence control | AMOT | Xq23 | 4 | 100 |
| Y presence control | UTY | Yq11.221 | 15 | 105 |
| reference probe | RAB7A | 03q21.3 | 3 | 130 |
| reference probe | DNAH5 | 05p15.2 | 2 | 134 |
| probe | RIPK2 | 08q21.3 | 3 | 139 |
| probe | PPP2R2A | 08p21.2 | 11 | 143 |
| reference probe | RPGRIP1 | 14q11.2 | 10 | 149 |
| probe | MCM7 | 07q22.1 | 11 | 154 |
| probe | ANXA7 | 10q22.2 | 4 | 160 |
| reference probe | CACNA1A | 19p13.2 | 27 | 166 |
| probe | NCOA2 | 08q13.3 | 8 | 172 |
| probe | APC | 05q22.2 | 4 | 178 |
| probe | TSG1 | 06q16.1 | 3 | 183 |
| probe | MYC | 08q24.21 | 3 | 189 |
| probe | TCEB1 | 08q21.11 | 6 | 196 |
| reference probe | CACNA1S | 01q32.1 | 8 | 202 |
| probe | MIR151A | 08q24.3 | - | 208 |
| probe | MEF2C | 05q14.3 | 4 | 214 |
| probe | EZH2 | 07q36.1 | 3 | 220 |
| probe | VCL | 10q22.2 | 22 | 226 |
| probe | WRB | 21q22.2 | 3 | 232 |
| reference probe | GLRB | 04q32.1 | 11 | 236 |
| probe | TNFRSF10B | 08p21.3 | 5b | 244 |
| probe | MXI1 | 10q25.2 | 8 | 250 |
| probe | MIR15A | 13q14.2 | 1 | 256 |
| probe | PIK3CA | 03q26.32 | 7 | 262 |
| probe | FYN | 06q21 | 10 | 266 |
| probe | DSCAM | 21q22.2 | 4 | 274 |
| probe | AR | Xq12 | 5 | 279 |
| reference probe | ALDOB | 09q31.1 | 3 | 285 |
| probe | ETS2 | 21q22.2 | 3 | 292 |
| probe | CDH1 | 16q22.1 | 14 | 297 |
| reference probe | COL11A1 | 01p21.1 | 11 | 301 |
| probe | AR | Xq12 | 1b | 309 |
| probe | ZFHX3 | 16q22.3 | 5 | 315 |
| probe | EZH2 | 07q36.1 | 9b | 322 |
| probe | ERG | 21q22.2 | 4 | 332 |
| probe | KLF5 | 13q22.1 | 2 | 337 |
| probe | NKX3-1 | 08p21.2 | 2 | 346 |
| probe | PTEN | 10q23.31 | 8 | 355 |
| probe | KCNMA1 | 10q22.3 | 30a | 361 |
| reference probe | TOP2A | 17q21.2 | 14 | 369 |
| probe | TMPRSS2 | 21q22.3 | 6 | 377 |
| probe | WWOX | 16q23.1 | 7 | 385 |
| probe | KIAA0196 | 08q24.13 | 2 | 394 |
| reference probe | COL3A1 | 02q32.2 | 17 | 400 |
| probe | TMPRSS2 | 21q22.3 | 14 | 409 |
| probe | FOXO1 | 13q14.11 | 2 | 418 |
| probe | RAD21 | 08q24.11 | 10 | 427 |
| probe | PIK3CA | 03q26.32 | 2 | 434 |
| probe | BACE2 | 21q22.2 | 2 | 443 |
| reference probe | OPA1 | 03q29 | 11 | 450 |
| probe | ERG | 21q22.2 | 12 | 457 |
| probe | APC | 05q22.2 | 10 | 463 |
| probe | RB1 | 13q14.2 | 27 | 472 |
| probe | PTEN | 10q23.31 | 4 | 481 |
| reference probe | SPG11 | 15q21.1 | 11 | 490 |
| reference probe | SMPD1 | 11p15.4 | 2a | 500 |

Q-64, Q-70, Q-76, Q-82: Q-fragments as controls for DNA quantity, only visible with less than 100 ng sample DNA.

CARM1 (88), IL1B (92), JPH3 (96): Low signals of the D-fragments indicate incomplete denaturation/ligation.

X presence control (100), Y presence control (105): Specific for the X or Y chromosome.

13 reference probes:

43 probes: genes to be examined for copy number variation.
